# The rhythmic expression of clock genes attenuated in human plaque-derived vascular smooth muscle cells

**DOI:** 10.1186/1476-511X-13-14

**Published:** 2014-01-13

**Authors:** Changpo Lin, Xiao Tang, Zhu Zhu, Xiaohong Liao, Ran Zhao, Weiguo Fu, Bin Chen, Junhao Jiang, Ruizhe Qian, Daqiao Guo

**Affiliations:** 1 Institute of Vascular Surgery, Department of Vascular Surgery, Zhongshan Hospital, Fudan University, Shanghai 200032, China; 2 Department of Physiology and Pathophysiology, Fudan University Shanghai Medical College, Shanghai 200032, China

**Keywords:** Circadian rhythm, Primary cell culture, Human vascular smooth muscle cells, Atherosclerosis, Plaque rupture

## Abstract

**Background:**

Acute myocardial infarction and stroke are more likely to occur in the early morning. Circadian pacemakers are considered to be involved in the process. Many peripheral tissues and cells also contain clock systems. In this study, we examined whether the primary cultured human plaque-derived vascular smooth muscle cells (VSMCs) process circadian rhythmicity; furthermore, we investigated the expression difference of clock genes between normal human carotid VSMCs and human plaque-derived VSMCs.

**Methods:**

Fifty-six human carotid plaques provided the atherosclerotic tissue, and 21 samples yielded viable cultured primary VSMCs. The normal carotid VSMCs were cultured from donors’ normal carotids. The mRNA levels of the target genes were measured by Quantitative Real-Time Polymerase Chain Reaction (qRT-PCR).

**Results:**

After serum shock, both types of cells showed clear circadian expressions of Bmal1, Cry1, Cry2, Per1, Per2, Per3 and Rev-erbα mRNA; meanwhile the Clock mRNA show a rhythmic expression in plaque-derived SMCs but not in normal carotid VSMCs. The expression levels of these main clock genes were significantly attenuated in human plaque-derived VSMCs compared with normal human carotid VSMCs. The rhythm of Bmal1 mRNA in plaque-derived VSMCs was changed.

**Conclusion:**

The present results demonstrate that the human plaque-derived VSMCs possess different circadian rhythmicity from that of normal carotid VSMCs. The rhythm changes of clock genes in plaque-derived VSMCs may be involved in the process of atherosclerosis and finally promote the rupture of plaque.

## Background

In mammals, many behavioral and physiological processes exhibit circadian (approximately 24 h) rhythms that are controlled by a clock system. This system includes the central circadian clock residing in the hypothalamic suprachiasmatic nucleus (SCN) [1] and the peripheral clock located in many peripheral tissues. It is considered that circadian rhythmicity of peripheral tissues is uniquely controlled by SCN via neural and humoral signals. However, recent research demonstrates that peripheral tissues and cells also contain a similar clock system to that in the SCN [2,3]. The core clock genes include Bmal1, Clock, Cry, Per and Rev-erbα etc., which form a negative feedback loop involving a positive limb (Bmal1 and Clock) and a negative limb (Per and Cry) [4]. The heterodimer of BMAL1/CLOCK binds to the E-boxes located within the promoters of Cry and Per genes and activates their transcription. Then, the proteins of PER and CRY form a complex and inhibit the positive limb, resulting in rhythmic oscillation.

Acute myocardial infarction and stroke, severe complications resulted from atherosclerosis, are more likely to occur in the early morning [5,6]. These fatal complications of atherosclerosis are mainly caused by plaque rupture and subsequent embolism and thrombosis. Epidemiological studies have also indicated that shift workers suffer from a higher risk of atherosclerosis and cardiovascular events [7]. These phenomena cannot simply be explained by the change in blood pressure and platelet function [8]. Although the underlying molecular mechanisms of such diurnal variations were not clarified, the circadian clock could be a potential factor involved in the process. Previous animal research has already illustrated that the disruption of circadian rhythms could impair vessels and enhance atherosclerosis [9,10].

Vascular smooth muscle cells (VSMCs) are responsible for the structure and function of vessel walls and are involved in the development and progression of a variety of cardiovascular diseases, such as atherosclerosis [11]. However, little is known about the circadian clock system in human VSMCs, especially the VSMCs in human plaques. In the present study, we established a model of primary cultured human plaque-derived VSMCs and normal human carotid VSMCs in vitro (both possess circadian oscillators by the serum shock method) to compare the rhythm changes of clock genes in human plaque-derived VSMCs with that in normal human carotid VSMCs.

## Results

### Primary cultured VSMCs

**Figure 1:**
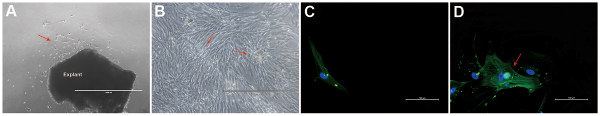
**The morphologies and immunofluorescence analysis of VMSCs cultured from human plaque. (A)** Cells starting to grow out radially from the explants within 7 to 12 days (red arrows). **(B)** Cells growing into cells and showing typical “hills and valleys” morphology in about 4 weeks. Larger “hills” form into nodules (red arrows); **(C)** α SMA expression in human plaque-derived VSMCs with a fusiform shape; cytoplasm (green), nucleus location (blue). **(D)** Uniform filamentous of α-SMA (red arrows) can be observed universally in human plaque-derived VSMCs with a big, flattened shape.

Fifty-six patients underwent carotid endarterectomy between May 2012 and July 2013 in Zhongshan Hospital (Shanghai, China), and 21 of them were successfully cultured plaque-derived VSMCs. Seven of the total 10 donors yielded viable cultured normal VSMCs. More demographics and characteristics of patients and donors are summarized in Table 1. Cells started to migrate from the explanted tissues within 7 to 12 days (Figure 1A) and formed typical “hills and valleys” in about 4 weeks (Figure 1B).

**Table 1:** Main characteristics of patients succeeded in culturing VSMCs

	**Number of cases**	**Gender (M/F)**	**Age range (mean)**	**Hypertension**	**Hyperlipidemia**	**DM**
**Type**						
Human plaque derive VSMCs	21	14/7	55-82 (69)	19	1	7
Normal human carotid VSMCs	7	4/3	29-66 (44)	2	0	0

M, male; F, female; DM, diabetes mellitus.

**Figure 2:**
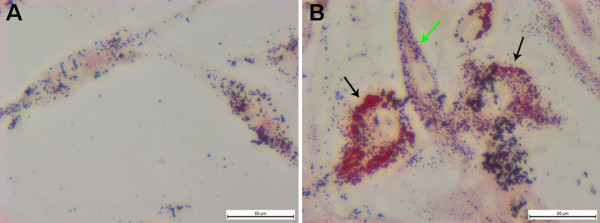
**Oil Red O staining of human primary cultured VSMCs. (A)** Oil Red O staining of normal human carotid VSMCs after 7 weeks in the 3^rd^ passage. **(B)** Oil Red O staining of human plaque-derived VSMCs with two distinctly different morphologies after 7 weeks in the 3^rd^ passage. The big, flattened shape ones (black arrow) contain much more lipid than the fusiform ones (green arrow).

**Figure 3:**
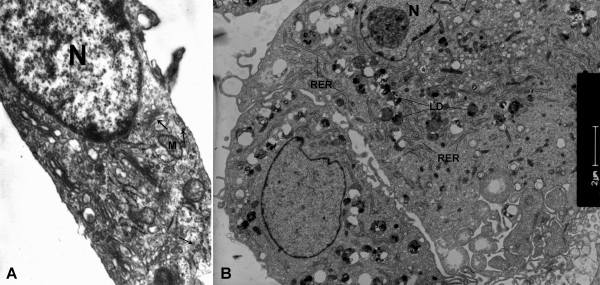
**Transmission electron micrograph of human plaque-derived VSMCs cultured for 7 weeks in the 3**^**rd **^**passage. (A)** The ultrastructure of fusiform plaque-derived VSMCs. Arrows show myofilaments with dense bodies. ×6000. **(B)** The ultrastructure of human plaque-derived VSMCs with a big, flattened shape. N: nucleus; M: mitochondria; RER: rough endoplasmic reticulum; LD: lipid droplets.

The morphologies of the two sources of cells were quite different. VSMCs cultured from normal carotid had a typical fusiform shape, while cells cultured from plaques had two distinctly different morphologies: fusiform and a big, flattened shape (Figure 1C and D). And the big, flattened shape ones accounted for the majority of human plaque-derived VSMCs. The VSMC marker, smooth muscle cell protein α - smooth muscle actin (α SMA), was expressed in all phenotypes (Figure 1C and D). But the Oil-red- O staining demonstrated that the lipid content within human plaque-derived VSMCs was much richer than that in normal human carotid VSMCs (Figure 2). And beside the different shapes observed in human plaque-derived VSMCs, the cells with a big, flattened shape contain much more lipid than the fusiform ones, whose lipid content was quite similar to normal human carotid VSMCs. Transmission electron microscopy revealed the fusiform ones had abundant myofilament bundles and distinct dense bodies (Figure 3A), while the cells with a big, flattened shape were full of rough endoplasmic reticulum (RER) and large lipid droplets (Figure 3B).

### Diurnal expression patterns of circadian genes in normal human carotid VSMCs

**Figure 4:**
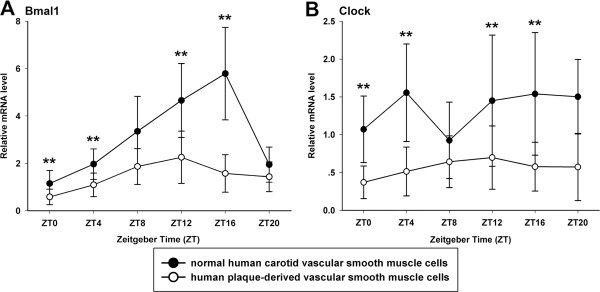
**Circadian expression of Bmal1 and Clock genes at mRNA levels in primary cultured VSMCs. (A)** Circadian expression of Bmal1 mRNA in normal human carotid VSMCs and human plaque-derived VSMCs. **(B)** Circadian expression of Clock mRNA in normal human carotid VSMCs and human plaque-derived VSMCs. The mRNA levels were determined by qRT-PCR at the indicated time points after the serum shock. Values of Bmal1 and Clock mRNA were normalized to GAPDH mRNA. The signal levels at ZT0 of normal human carotid VSMCs were defined as 1. Each value represents the mean ± SD (n_1_ = 7 of normal human carotid VSMCs; n_2_ = 21 of human plaque-derived VSMCs). The differences of expression levels were assessed by unpaired Student’s t test. **p < 0.01 in normal human carotid VSMCs versus human plaque-derived VSMCs.

**Figure 5:**
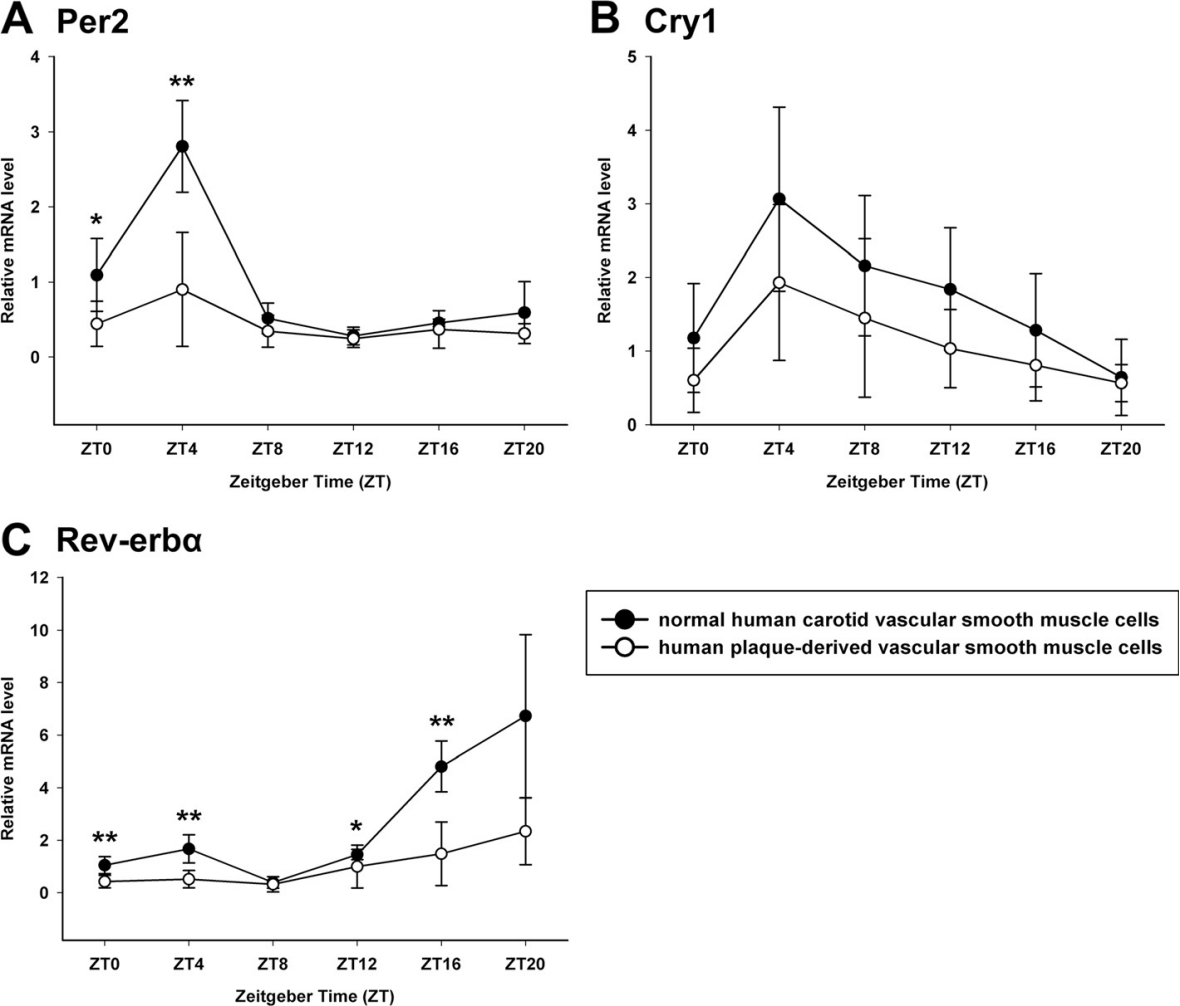
**Circadian expression of Per2, Cry1 and Rev-erbα at mRNA levels in primary cultured VSMCs. (A)** Circadian expression of Per2 mRNA in normal human carotid VSMCs and human plaque-derived VSMCs. **(B)** Circadian expression of Cry1 mRNA in normal human carotid VSMCs and human plaque-derived VSMCs. **(C)** Circadian expression of Rev-erbα mRNA in normal human carotid VSMCs and human plaque-derived VSMCs. The mRNA levels were determined by qRT-PCR at the indicated time points after the serum shock. Values of Per2, Cry1 and Rev-erbα mRNA were normalized to GAPDH mRNA. The signal levels at ZT0 of normal human carotid VSMCs were defined as 1. Each value represents the mean ± SD (n_1_ = 7 of normal human carotid VSMCs; n_2_ = 21 of human plaque-derived VSMCs). The differences of expression levels were assessed by unpaired Student’s t test. *p < 0.05 in normal human carotid VSMCs versus human plaque-derived VSMCs. **p < 0.01 in normal human carotid VSMCs versus human plaque-derived VSMCs.

**Figure 6:**
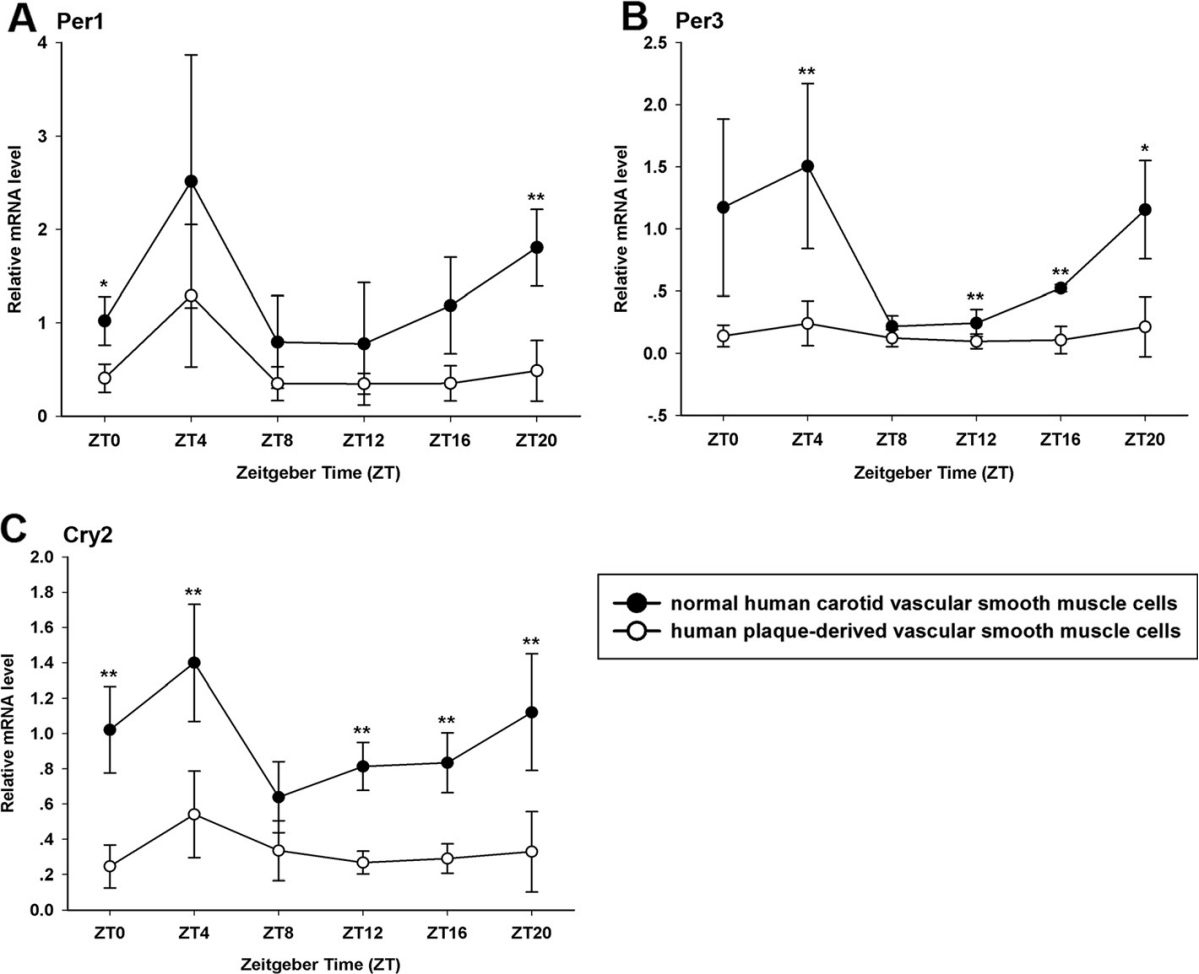
**Circadian expression of Per1, Per3 and Cry2 at mRNA levels in primary cultured VSMCs. (A)** Circadian expression of Per1 mRNA in normal human carotid VSMCs and human plaque-derived VSMCs. **(B)** Circadian expression of Per3 mRNA in normal human carotid VSMCs and human plaque-derived VSMCs. **(C)** Circadian expression of Cry2 mRNA in normal human carotid VSMCs and human plaque-derived VSMCs. The mRNA levels were determined by qRT-PCR at the indicated time points after the serum shock. Values of Per1, Per3,and Cry2 mRNA were normalized to GAPDH mRNA. The signal levels at ZT0 of normal human carotid VSMCs were defined as 1. Each value represents the mean ± SD (n_1_ = 3 of normal human carotid VSMCs; n_2_ = 5 of human plaque-derived VSMCs). The differences of expression levels were assessed by unpaired Student’s t test. *p < 0.05 in normal human carotid VSMCs versus human plaque-derived VSMCs. **p < 0.01 in normal human carotid VSMCs versus human plaque-derived VSMCs.

First, we detected whether VSMCs, which were induced from normal human carotids, presented cyclical rhythms. After being treated with serum shock (2 h), the core clock genes of Bmal1, Per1, Per2, Per3, Cry1, Cry2 and Rev-erbα mRNA had shown a 24-hour rhythmic oscillation in the cells (p < 0.05), while Clock mRNA did not show a rhythmicity (p > 0.05, Figure 4B). As illustrated in Figure 4A, the expression of Bmal1 mRNA peaked at ZT16 and had its lowest level at ZT0. The mRNA level of Per2 had a peak at ZT4 and a trough at ZT12 (Figure 5A). Meanwhile, the circadian rhythm of Cry1 mRNA, whose protein combined and formed heterodimer with the product of Per2, is therefore quite similar to that of Per2, with a peak at ZT4 and a trough at ZT20 (Figure 5B). The rhythmic expression of Per1, Per3 and Cry2 also peaked at ZT4 (Figure 6). The mRNA level of Rev-erbα was the lowest at ZT8 and peaked at ZT20 (Figure 5C).

### Expressions of clock genes attenuated in human plaque-derived VSMCs

We then detected that the human plaque-derived VSMCs also possess circadian oscillators (p < 0.05), and the rhythms of their core clock genes expression were similar to that of normal human carotid VSMCs, with consistent peak and trough times (except Bmal1 and Clock) (Figures 4, 5 and 6). Compared with normal human carotid VSMCs, the peak mRNA level of Bmal1 phase advancing to ZT12 in human plaque-derived VSMCs. Interestingly, the expression of the Clock gene also showed a rhythmic oscillation in plaque-derived SMCs (p < 0.05), which was different to normal human carotid VSMCs (p > 0.05), with a peak at ZT12. However, the amplitude of the above genes was significantly attenuated in human plaque-derived VSMCs compared with normal human carotid VSMCs.

## Discussion

VSMCs are the major cell type in vessel walls and are responsible for the structure and function of vessel walls. They are involved in the pathogenesis of atherogenesis and plaque rupture. Previous research studies indicated that the VSMC phenotype switched in atherosclerosis [12,13], which is consistent with our findings. VSMCs derived from human plaques can be divided into two distinctly different phenotypes. According to their morphologies, content of lipid and ultrastructure, the fusiform cells would be the contractile VSMCs, and the phenotype of big shape ones was switching to the synthetic type. This phenomenon implied that VSMCs may play different roles at distinct stages of atherogenesis.

Both peripheral tissues and cells in vivo or in vitro possess circadian oscillators, which is similar to that in the SCN. The different cellular elements of vasculature, including vascular endothelial, smooth muscle, and fibroblasts cells, have proved to show a rhythm expression of circadian genes in vitro. The cultured fibroblasts, as well as the hemangioendothelioma cells in culture, present a circadian expression for all clock genes after treatment with serum shock [14,15]. Moreover, the rhythmic pattern of expression in cultured fibroblasts persists for over 20 days, suggesting the clock system is self-sustained [16]. Several studies have demonstrated that molecular oscillators exist in either mouse or human aortic VSMCs in vitro, and the rhythms are distinct between different species or vessels [17-19].

**Figure 7:**
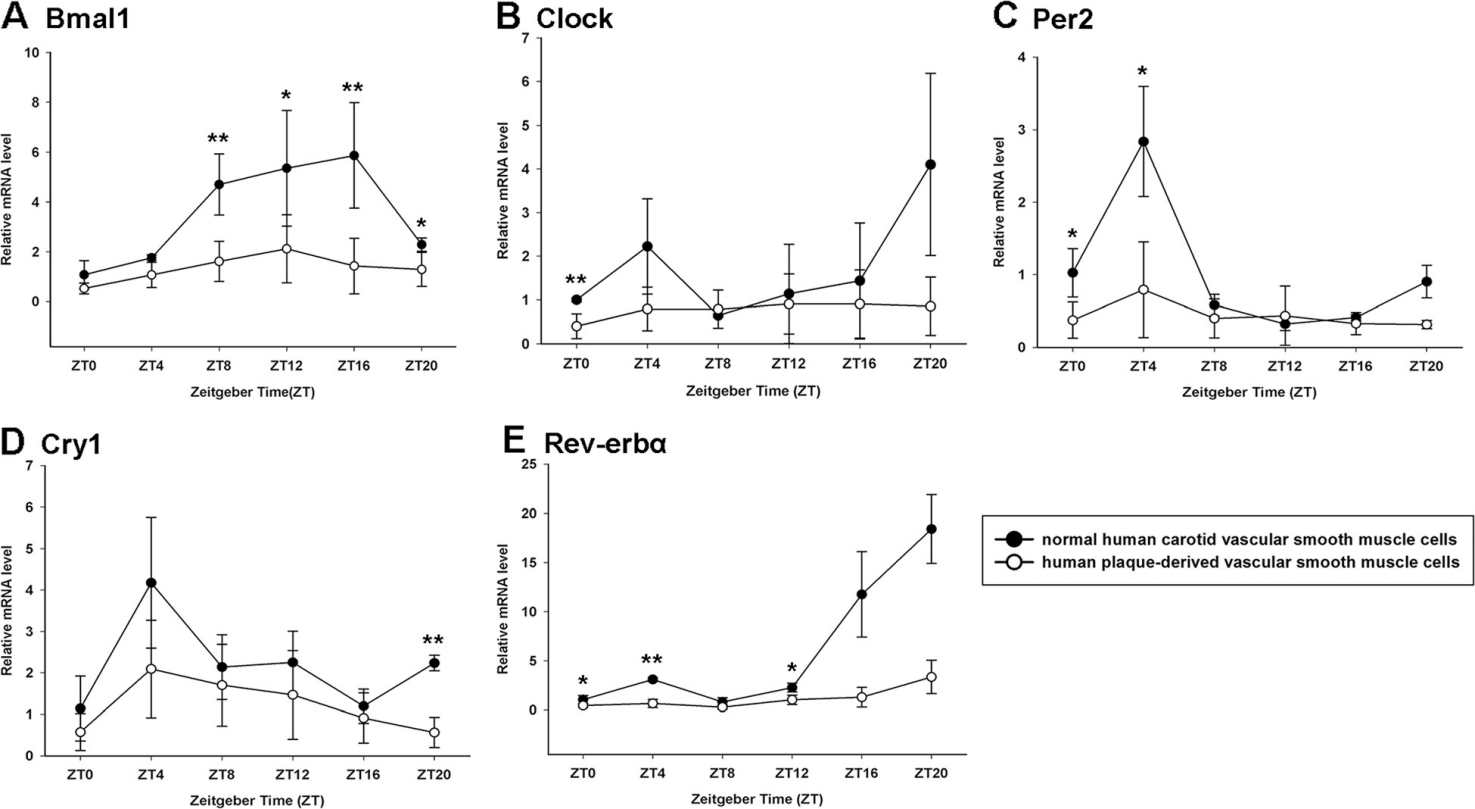
**Subgroup comparison of circadian expression of clock genes at mRNA levels between two groups according to approximate age (range of age: 60–70 years old). (A)** Circadian expression of Bmal1 mRNA in two subgroups. **(B)** Circadian expression of Clock mRNA in two subgroups. **(C)** Circadian expression of Per2 mRNA in two subgroups. **(D)** Circadian expression of Cry1 mRNA in two subgroups. **(E)** Circadian expression of Rev-erbα mRNA in two subgroups. The mRNA levels were determined by qRT-PCR at the indicated time points after the serum shock. Values of clock genes mRNA were normalized to GAPDH mRNA. The signal levels at ZT0 of normal human carotid VSMCs were defined as 1. Each value represents the mean ± SD (n_1_ = 2 of normal human carotid VSMCs; n_2_ = 6 of human plaque-derived VSMCs). The differences of expression levels were assessed by unpaired Student’s t test. *p < 0.05 in normal human carotid VSMCs versus human plaque-derived VSMCs. **p < 0.01 in normal human carotid VSMCs versus human plaque-derived VSMCs.

In the present study, we found that the human plaque-derived VSMCs and normal human carotid VSMCs in vitro also possess the rhythmic oscillation of clock genes. The main clock genes, including Bmal1, Per, Cry and Rev-erbα, showed a similar expression rhythm between the two groups in our experiments. But the Clock gene showed a rhythmic expression in plaque-derived SMCs, which was no significant rhythmicity in normal human carotid VSMCs. This result was different from the previous animal experiment, where the Clock gene did not show a rhythmic oscillation in mice [20]. Therefore, it required a larger sample size to confirm in future studies. Interestingly, the expression levels and oscillation amplitude of these genes were significantly attenuated in human plaque-derived VSMCs compared with normal human carotid VSMCs. As aging could lead to impairment and disruption in circadian rhythmicity, we conducted a subgroup comparison of approximate age (range of age: 60–70 years old; Table 2). We found the expression levels of clock genes were still significant higher in normal human carotid SMCs than in human plaque-derived VSMCs (Figure 7). So we infered the attenuation of expression is partly caused by the senescence of VSMCs in atherosclerosis. It was observed that human plaque–derived VSMCs illustrated numerous features of senescence [21]: (1) restricted proliferative capacity, (2) high senescence-associated β-galactosidase (SAβG) activity even at early stages of culture, (3) significantly reduced percentage of S phase cells and increased percentage of cells in G1, and (4) telomere shortening compared with VSMCs derived from normal vessels. Illi et al. [22] demonstrated that clock gene expressions in senescent VSMCs were attenuated compared with young counterparts. They believe it is related, at least in part, to impaired CREB activation and telomeres shortening.

**Table 2:** Main characteristics of patients involved in subgroup analysis (range of age: 60–70 years old)

	**Number of cases**	**Gender (M/F)**	**Age range (mean)**	**Hypertension**	**Hyperlipidemia**	**DM**
**Type**						
Human plaque derive VSMCs	6	5/1	60-67 (63)	4	0	0
Normal human carotid VSMCs	2	1/1	64-66 (65)	2	0	0

M, male; F, female; DM, diabetes mellitus.

Interestingly, we previously found that in apolipoprotein E knockout mice (a widely used atherosclerotic mouse model), the expression levels and the circadian rhythms of clock genes changed compared to C57BL/6 J mice, and these changes were accompanied with a progression of atherosclerosis [20]. Pan, Jiang and Hussain [9] found that the mutation of Clock protein can impair cholesterol metabolism and enhance atherosclerosis in different mouse models. Anea et al. [10] illustrated that in mice with aberrant circadian rhythms, Clock mutant and Bmal1-knockout, pathological remodeling and vascular injury (such as intimal hyperplasia) increased. Moreover, the aged Bmal1-knockout mice exhibit even more severe abnormalities and a significant susceptibility to thrombosis. According to our results, the expressions of clock genes are significant decreased in human plaque-derived VSMCs compared to normal human carotid VSMCs. We hypothesized that the impairment of circadian rhythms in human plaque-derived VSMCs could promote the progress of atherosclerosis. As the plaque VSMCs in our experiment were derived from aged patients, we assumed that these patients with impaired circadian rhythms may also have been more susceptibility to thrombosis and subsequent cardiovascular events. Impressively, besides the reduction of amplitude, we found that the peak time of Bmal1 rhythmic oscillation was also changed. It was a limitation of this study that a certain cause was not found. Whether the change of Bmal1 rhythm is essential to the function of the clock system is also unknown and needs further in-depth studies.

In summary, we found that both the human plaque-derived VSMCs and normal carotid VSMCs possess circadian clock systems. The levels and rhythms of the core clock genes’ expression were changed in plaque-derived VSMCs compared with normal human carotid VSMCs, and these changes together may be involved in the progression of atherosclerosis and its subsequent complications. Of course, further research should be conducted to detect the protein expression of clock genes considered, and to find the downstream genes whose expressions are controlled by clock genes and to reveal how circadian genes regulate these clock-controlled genes and affect the diurnal variations of cardiovascular function.

## Materials and methods

### Human primary VSMCs culture

We used the established explant culture method to obtain human VSMCs [23]. Human plaque VSMCs were cultured from carotid plaques of patients who had undergone carotid endarterectomy. Donors from the Zhongshan Hospital Transplant Program provided the sections of carotid to culture the normal human carotid VSMCs. Tissues were minced into approximately 1 × 1-mm pieces and then carefully placed into T-25 flasks (NUNC) and cultured in the complete medium involving medium 199 (GIBCO) with 20% fetal bovine serum (GIBCO) and antibiotics (100 IU/ml penicillin, 100 mg/ml streptomycin and 250 ng/ml Amphotericin B). The cultures were incubated at 37°C, with the medium changed twice a week. For the passaging culture, cells were trypsinised with 0.25% trypsin (GIBCO) for approximately 3 min, then centrifuged at 200 × g for 5 min, resuspended, and split 1:2. The second passages of cells were seeded into 35-mm petri dishes for harvesting.

### Immunofluorescence microscopic detection of α - smooth muscle actin (α SMA) in primary cultured human VSMCs

Human VSMCs were cultured in collagen-I coated glass bottom dishes. Cells were washed softly and fixed with 4% paraformaldehyde for 10 min at room temperature and then rinsed with PBS (5 min×3 times). Subsequently, cells were permeated with 0.5% Triton-100 for 15 min and washed with PBS (5 min×3 times) before incubating with 1% normal donkey serum in PBS for 30 min. Then, cells were incubated with α - smooth muscle actin (a-SMA) antibody (1:100, BOSTER, CHINA) overnight at 4°C. Cells were washed with PBS (3 min×3 times), and the primary antibody was bound with FITC-labeled donkey anti-rat IgG for 60 min at 37°C. After washing with PBS (3 min×3 times), the cells were viewed through a Zeiss LSM 510 Meta confocal microscope.

### Oil Red O staining

Vascular smooth muscle cells were fixed with 4% paraformaldehyde for 20 min and washed with PBS. Fixed cells were stained with freshly prepared Oil Red O working solution (60% Oil Red O stock solution diluted by distilled water) for 30 min. Stained cells were rinsed with PBS until the background became clear, and then observed using an inverted microscope.

### Transmission Electron Microscope (TEM)

Vascular smooth muscle cells were centrifuged at 1500×g for 15 min, fixed with 2.5% glutaraldehyde, followed by postfixation for 2 h in 1% osmium tetroxide, dehydrated in graded alcohols and acetones. After that, the samples were embedded in Epon 812, and sectioned with LKB-I ultramicrotome in 50–60 nm. Then the sections were stained with 3% uranyl acetate and lead citrate, and examined by TEM (PHILIPS CM-120).

### Serum shock and cells harvesting

The serum shock was performed before cell harvesting, as described [24]. Briefly, human VSMC was grown in the complete medium for 72 h. Then, cells were starved for 24 h in serum-free medium 199 containing antibiotics (the same levels as the above). Subsequently (on the day of serum shock), cells were treated with medium 199 containing 50% horse serum for 2 h (serum shock) and then changed back to a starvation medium until the end of the experiment. The timing of the beginning serum shock was defined as Zeitgeber time 0 (ZT0), and cells were harvested for RNA extraction at ZT0, ZT4, ZT8, ZT12, ZT16 and ZT20.

### Total RNA isolation and complementary DNA preparation

The total RNA was extracted from harvested VSMCs using TRIzol reagent (Invitrogen Corporation, USA). First-strand cDNA was synthesized and amplified from 2 μg of total RNA using the ReverTra Ace qPCR RT Kit (TOYOBO, Japan).

### Quantitative real-time PCR

The messenger RNA (mRNA) levels of the target genes were measured by quantitative real-time PCR (iCyler iQ Real-time PCR Detection System, Bio-Rad Laboratories Inc, USA) using SYBR Green Real-time PCR Master Mix (Bio-Rad) in a total volume of 20 μl. All the samples were assayed in one essay in our study. The relative quantification of gene expression was analyzed from the measured threshold cycles (*C*_T_) by using the 2-ΔΔCt method in the experiment [25]. Glyceraldehyde-3-phosphate dehydrogenase (GAPDH) was used as an internal standard to normalize the expression level of each mRNA. Primers were designed by PRIMER 5.0. The target gene names and their primer sequences are shown in Table 3.

**Table 3:** The primer sequences used for PCR amplification

**Gene**	**GenBank accession**	**Forward primer (5′–3′)**	**Reverse primer (5′–3′)**
Bmal1	NM_001030272	TGGATGAAGACAACGAACCA	TAGCTGTTGCCCTCTGGTCT
Clock	NM_001267843	CAGAGCACCTTCCCTCAGTC	TTTCCCTCCTTTCCTCAGGT
Per1	NM_002616.2	TTCCTGACGGGCCGAAT	CGCTTGCAACGCAGCA
Per2	NM_022817	CGTGCCAAGCAGTTGACTTA	CAGCAAGGCTCAACAAATCA
Per3	NM_016831.1	GGTCGGGCATAAGCCAATG	GTGTTTAAATTCTTCCGAGGTCAAA
Cry1	NM_004075	TAAGAGGCTTCCCTGCAAAA	GCCTCCATTCCCATTAGGAT
Cry2	NM_001127457	AGGAGAACCACGACGAGA	TCCGCTTCACCTTTTTATAC
Rev-erbα	NM_021724	CTGGGAGGATTTCTCCATGA	TCACTGTCTGGTCCTTCACG
GAPDH	NM_001256799	GTCAGTGGTGGACCTGACCT	TGCTGTAGCCAAATTCGTTG

### Statistical analysis

SPSS 19.0 software was used to perform the statistical analysis. Results were demonstrated as mean ± SEM. The values for mRNA levels were presented as relative values in all experiments. An unpaired Student’s t test was conducted to examine the differences between the groups, and a two-way analysis of variance (ANOVA) was used to evaluate the oscillation of each gene expression. In all the analyses, p < 0.05 was considered statistically significant.

### Ethical statement

The study protocol was conducted following the principles outlined in the Declaration of Helsinki and approved by the Ethics Committee of the Zhongshan Hospital, Fudan University. All human tissues were collected from patients who signed the informed consent. And all normal donors signed the consent voluntary standing on altruism. We also obtained the permissions from their immediate family members. No donor organs were obtained from executed prisoners or other institutionalized persons.

## Abbreviations

VSMCs: Vascular smooth muscle cells; SCN: Suprachiasmatic nucleus; Bmal1: Brain and muscle Arnt-like 1; Clock: Circadian locomotor output cycles kaput; Per: Period; Cry: Cryptochrome; Rev-erbα: NR1D1 (nuclear receptor subfamily 1, group D, member 1) a member of the nuclear receptor family of intracellular transcription factors; GAPDH: Glyceraldehyde-3-phosphate dehydrogenase; TEM: Transmission Electron Microscope; N: Nucleus; M: Mitochondria; RER: Rough endoplasmic reticulum; LD: Lipid droplets.

## Competing interests

The authors declare that they have no competing interests.

## Authors’ contributions

CL (Changpo Lin) and XT (Xiao Tang) carried out all aspects of experiments and data analysis, and drafted the manuscript. ZZ, XL participated in the figure formatting and RZ performed the statistical analysis. WF, BC and JJ participated in the design of experiments. RQ (Ruizhe Qian) participated in the design of study and proofread manuscript. DG (Daqiao Guo) conceived of the study and performed the experimental instruction. All authors read and approved the final manuscript.
